# The genetics of a putative social trait in natural populations of yeast

**DOI:** 10.1111/mec.12904

**Published:** 2014-10-04

**Authors:** G O Bozdag, D Greig

**Affiliations:** * Max Planck Institute for Evolutionary BiologyAugust Thienemann Strasse 2, Plön, 24306, Germany; † The Galton Laboratory, Department of Genetics, Evolution, and Environment, University College LondonGower Street, London, WC1E 6BT, UK

**Keywords:** cheating, cooperation, copy number variation, droplet digital PCR, *Saccharomyces*, *
SUC
*

## Abstract

The sharing of secreted invertase by yeast cells is a well-established laboratory model for cooperation, but the only evidence that such cooperation occurs in nature is that the *SUC* loci, which encode invertase, vary in number and functionality. Genotypes that do not produce invertase can act as ‘cheats’ in laboratory experiments, growing on the glucose that is released when invertase producers, or ‘cooperators’, digest sucrose. However, genetic variation for invertase production might instead be explained by adaptation of different populations to different local availabilities of sucrose, the substrate for invertase. Here we find that 110 wild yeast strains isolated from natural habitats, and all contained a single *SUC* locus and produced invertase; none were ‘cheats’. The only genetic variants we found were three strains isolated instead from sucrose-rich nectar, which produced higher levels of invertase from three additional *SUC* loci at their subtelomeres. We argue that the pattern of *SUC* gene variation is better explained by local adaptation than by social conflict.

## Introduction

In contrast to other eukaryotes, the genome of *Saccharomyces cerevisiae* is compact, containing few redundant genes or pseudogenes (Goffeau *et al.* 1996; Lafontaine *et al.* 2004). The *SUC* genes, which encode the extracellular enzyme invertase, are exceptional. There are nine known loci for *SUC* genes: *SUC1*–*SUC5* and *SUC7*–*SUC10* (Naumov & Naumova 2010). *SUC2*, the ancestral locus, is located in the left arm of chromosome IX, but the other copies are all found at subtelomeric regions (Carlson & Botstein 1983; Carlson *et al.* 1985; Naumov & Naumova 2010). Most strains contain only a single *SUC2* gene, but some contain one or more of the subtelomeric *SUC* loci in addition to *SUC2*, and others have a *suc2* pseudogene and produce no invertase (Carlson & Botstein 1983; Naumov *et al.* 1996; Denayrolles *et al.* 1997).

The variation in *SUC* genotypes can be explained using social evolution theory (Greig & Travisano 2004). The invertase produced from *SUC* genes is secreted to digest extracellular sucrose into the preferred sugars glucose and fructose, which can be taken up by the cell and metabolized. Sugars diffuse readily, so cells that cannot produce invertase themselves because they lack any functional *SUC* genes can use the glucose and fructose produced by those that do (Gore *et al.* 2009). Thus, invertase production is analogous to public goods cooperation with nonproducers as cheats that can exploit and invade populations of cooperators (Greig & Travisano 2004). The phenomenon of telomeric silencing (Wyrick *et al.* 1999) has been used to explain subtelomeric *SUC* loci. If *suc2* cheats retain an unexpressed but functional copy of *SUC*, then having invaded a colony of cooperators and depleted the public good, they could regain the ability to produce invertase from a silent subtelomeric ‘backup’ copy (Greig & Travisano 2004). Consistent with this social theory model, laboratory experiments find that the relative fitness of nonproducers can be higher or lower than that of producers, depending on factors such as density (Greig & Travisano 2004), frequency (Gore *et al.* 2009; Damore & Gore 2012) and sucrose concentration (Koschwanez *et al.* 2011). However, a recent experiment found that mixed cultures of producers and nonproducers had higher mean fitness than monocultures of producers, inconsistent with the model of nonproducers as cheats (MacLean *et al.* 2010).

An alternative explanation for *SUC* variation is that different *SUC* genotypes have adapted to environments with different availabilities of sucrose. For thousands of years, humans have used yeast to make alcohol, and more recently, to raise bread, to flavour foods, to study genetics and to secrete bio-engineered products such as insulin (Thim *et al.* 1986; Botstein & Fink 1988; Porro *et al.* 1995). A survey of the drinks available in a typical bar reveals some of the diverse substrates that domesticated yeast strains are grown on. Yeast produces invertase constitutively, even in the absence of sucrose, although high levels of glucose can suppress invertase production (MacLean *et al.* 2010). Substrates low in sucrose might favour the loss of costly invertase production and the selection of *suc2* null mutants. Conversely, substrates rich in sucrose might select for additional subtelomeric copies of *SUC* if they were not completely silenced and could therefore contributed to increased invertase production (Denayrolles *et al.* 1997; Batista *et al.* 2004). Thus, the observed diversity in *SUC* genotypes may simply be due to domestication in different environments (Libkind *et al.* 2011). Similar increases in diversity are seen in other domesticated species, for example domesticated dogs have much greater morphological variation than wolves, their wild ancestors (Wayne 1986) indeed the range of body sizes in among breeds of this single domesticated species exceeds the range of all other wild canid species (Lindblad-Toh *et al.* 2005).

These two competing hypotheses can be tested by examining how individuals vary within and between habitats. The social conflict hypothesis predicts that different strains isolated from the same habitat will differ in their *SUC* genotypes, because some will be cheats and others will be cooperators. The sucrose adaptation hypothesis predicts that different strains from the same habitat will have the same *SUC* genotype, but that strains from environments with different sucrose availabilities will differ. Naumov *et al*. (1996) surveyed *SUC* gene variation in 91 strains isolated from many different environments, finding eleven invertase nonproducing strains that contained only a nonfunctional *suc2* allele. Five of these came from olive processing (Vaughan & Martini 1987), and two came from human faeces (Naumov *et al.* 1990), environments that are low in sucrose (Marsilio *et al.* 2001), which is consistent with the sucrose adaptation hypothesis. But two came from wine, an environment that also provided many invertase producers, consistent with the social conflict hypothesis. The remaining nonproducer (GM51) has unknown origins (Naumov *et al.* 1996). Ten strains had multiple *SUC* genes, and all came from sucrose-rich environments (strawberry, grape, ginger wine, billi wine and palm wine, Naumov *et al.* 1993; Basson *et al.* 2010; Kim & Lee 2006) or from fermentations that are artificially supplemented with sucrose (distilling and champagne making, Naumov *et al.* 1996), supporting the sucrose adaptation hypothesis. These results are difficult to interpret because different lineages of *S. cerevisiae* are often genetically mixed (Liti *et al.* 2009), perhaps by the process of human domestication (Libkind *et al.* 2011), and because many of the strains were not systematically isolated and their origins are unclear.

We therefore decided to determine the frequency of invertase nonproducers in *S. paradoxus,* the wild relative to *S. cerevisiae. S. paradoxus* has several advantages over *S. cerevisiae* for this study. The most important is that *S. paradoxus* is not used in human fermentations and instead has a well-established and well-sampled natural habitat extending to several continents on oak trees (Naumov *et al.* 1998; Johnson *et al.* 2004), and on Canadian maple trees (Charron *et al.* 2014). Unlike *S. cerevisiae*, whose populations show little geographical structure and high gene flow, perhaps because humans move strains around the world and mix them (Liti *et al.* 2009), *S. paradoxus* populations have strong geographical structure, with little mixing between lineages from different places (Kuehne *et al.* 2007; Liti *et al.* 2009). These properties mean that any *S. paradoxus* strain is likely to have evolved in the environment from which it was isolated, and is very unlikely to be a recent immigrant adapted to a different environment or to contain genetic material from such an immigrant. To test the hypothesis that social conflict should produce *SUC* variation among individuals within a single type of habitat, we determined the invertase production, the *SUC* loci and the *SUC* gene copy number of a set of 80 *S. paradoxus* strains: 65 isolated from oak trees and 15 isolated from maple trees. We did not have similarly large sets of *S. cerevisiae* strains from well-defined habitats and cannot exclude the possibility that some wild-caught strains might originate from human fermentations, or be related to such feral escapees. Nevertheless, we also tested 30 *S. cerevisiae* strains we could find that were isolated from apparently natural sources, including 15 recent isolates from primeval forests, which form distinct lineages compared to all the other *S. cerevisiae* strains identified so far (Wang *et al.* 2012). Finally, we tested whether strains with *SUC2* deleted produced invertase from their subtelomeric *SUC* loci or whether they were ‘silent’.

## Materials and methods

All strains, their original strain numbers, references, details of their origins and inclusion in genome sequencing projects are described in Appendix S1 (Supporting information).

To determine whether *SUC* genotypic variation occurs within (rather than between) wild populations, it is necessary to have multiple examples of wild strains isolated from a single well-defined habitat. *S. paradoxus* is ideal for this because it is not domesticated and many strains have been systematically isolated from oak trees. We tested all the oak-associated strains that we could access, including 29 that we isolated ourselves in Germany, 25 from the United Kingdom, 7 from Russia, 3 from North America and 1 from Japan (see Appendix S1, Supporting information for details). More recently, Canadian maple trees have been identified as a habitat for *S. paradoxus*, and we included 15 strains of *S. paradoxus* isolated from Canadian maple trees (Charron *et al.* 2014). We tested all *S. paradoxus* strains that we could acquire, but we excluded single strains isolated from unique or poorly described habitats and those from insect vectors which might have fed on unknown substrates.

We were concerned that any *S. cerevisiae* strains we tested might have recently escaped from human fermentations, or might have been crossed to such feral strains. Further, the natural habitat of *S. cerevisiae* is less well established than that of *S. paradoxus*. We therefore focused primarily on *S. paradoxus*. However, a large set of *S. cerevisiae* has recently been isolated from primeval forests in China, far from human influence, and there is good evidence they represent a truly wild population (Wang *et al.* 2012). We were able to get hold of 15 of these strains to test (7 from rotten wood, 2 from soil, 2 from oak, 2 from beech and one each from persimmon and oriental raisin trees). The majority of other available *S. cerevisiae* strains have been isolated from human fermentations or associated places, such as vineyards and food processing facilities. However, we were able to find 15 additional *S. cerevisiae* strains from a variety of apparently natural habitats: 5 from oak, 3 from soil, 3 from Bertram palm nectar and one each from cactus, cactus fruit, fig and cocoa.

We also tested various *S. cerevisiae* strains as controls and for comparison purposes. Our standard control strains were C.Lab.1. and C.Lab.1.*suc2::KANMX*, are isogenic with strains that have been used as a ‘cooperators’ and ‘cheats’, respectively, in previous laboratory studies on cooperation (Greig & Travisano 2004; MacLean & Brandon 2008; Gore *et al.* 2009; MacLean *et al.* 2010). We included two domesticated strains, C.Ginger.wine and C.Billi.wine, as positive controls with known multiple *SUC* copies. Finally, we knocked out the *SUC2* loci from these two strains as well as from the three wild strains that turned out to have multiple *SUC* copies, creating five new strains: C.Ginger.wine.*suc2::NATMX,* C.Billi.wine.*suc2::NATMX*, C.Nectar.1.*suc2::NATMX*, C.Nectar.2.*suc2::NATMX* and C.Nectar.3.*suc2::NATMX*.

### Screening wild strains for invertase nonproducers

To determine which of our strains produced invertase, we used the Glucose (HK) Assay Kit (Sigma-Aldrich, St. Louis, MO, USA), which produces a colorimetric reaction in response to glucose. We calibrated the assay using known dilutions of purified invertase (Sigma-Aldrich). Twenty microlitre of each dilution was combined with 100 μL sodium acetate buffer (0.2 m, pH = 5.2), and 50 μL of 0.5 m sucrose added. The reaction was incubated at 37 °C for 20 min, then stopped by adding 300 μL of 0.2 m K_2_HPO_4_ and heating at 100 °C for 5 min. One-hundred and fifty microlitre of this reaction mixture was added to 1 mL glucose assay reagent provided by the kit, and the optical absorbance at 340 nm was determined after following the kit instructions. We found the assay gave a linear response between absorbances of 0.11 and 0.78 (Appendix S4, Supporting information).

We optimized the assay conditions using a laboratory strain, C.Lab.1., which produces invertase from a single *SUC2* locus and has been used as a ‘cooperator’ in previous work on sociality (Greig & Travisano 2004; MacLean & Brandon 2008; Gore *et al.* 2009; MacLean *et al.* 2010). Each strain was grown in 2 mL of YEPD (1% yeast extract, 2% peptone, 2% dextrose) overnight at 30 °C. We spun down 1 mL of the culture, washed it with 1 mL of sterile water and centrifuged again. The pellet was resuspended in 1 mL of 0.9% sterile saline, and 5 μL of the cell suspension was spotted onto a YEPS plate (1% yeast extract, 2% peptone, 2% sucrose and 2.5% agar) and incubated it for 2 days at 30 °C. The resulting colony was then resuspended in 5 mL of sterile water, and a 100 μL sample was spun down and washed twice, then resuspended in 50 μL of sterile water, combined with 100 μL sodium acetate buffer and 50 μL of 0.5 m sucrose, incubated at 37 °C for 20 min and stopped with 300 μL of 0.2 m K_2_HPO_4_ heating as described above. One-hundred microlitre of the reaction mixture was added to 1 mL glucose assay reagent, and the absorbance was read. We used the same method on the isogenic ‘cheat’ strain C.Lab.1.*suc2::KANMX*. Pilot experiments indicated that wild strains produced so much more invertase than the laboratory ‘cooperator’ strain C.Lab.1 that they saturated the assay, so we reduced the volume of the resuspended cells from 100 to 20 μL, making the suspension up to 100 μL with 80 μL of the nonproducer C.Lab.1.*suc2::KANMX* prepared in the same way. Measurements were then multiplied by five to correct for this dilution. We screened the invertase production of all 110 wild strains in this way (see Appendix S1, Supporting information).

### *SUC* alleles in whole genome sequences

Whole genome sequences were available for 29 *S. paradoxus* and 8 *S. cerevisiae* strains. For details of which strains had sequences, and where the sequences can be accessed, please see Appendix S1 (Supporting information). These genome sequences were used to determine whether a strain contained intact *SUC* open reading frames or *suc* pseudogenes. The nucleotide sequences of *SUC* genes that were identified in this way are listed in Appendices S5 and S6 (Supporting information).

### Southern blots for *SUC* loci

Whole genome sequences were not available for most of our strains, and even for the 29 *S. paradoxus* and 8 *S. cerevisiae* that had been sequenced, we could not reliably infer the *SUC* loci or copy numbers from the sequences because of the short reads and low sequencing coverage. Subtelomeric *SUC* genes are embedded in highly repetitive DNA which may not be properly assembled in genome sequencing projects. To determine the *SUC* loci in our wild strains, we therefore made Southern blots of whole-chromosome pulsed-field gels, and probed them with labelled *SUC2* fragments. We also included controls on the pulsed-field gels: the C.Lab.1.*suc2::KANMX* as nonproducer containing no known *SUC* genes, C.Lab.1 as a producer containing a single *SUC2* gene and the domesticated strains C.Ginger.wine and C.Billi.wine as positive controls previously identified as containing multiple *SUC* loci (Naumov *et al.* 1996). All strains are described in Appendix S1 (Supporting information).

We prepared chromosomal DNA plugs according to Carle & Olson (1985). *S. cerevisiae* CHEF DNA size standard (YNN295 strain) was used in all pulse-field gel electrophoresis runs (Bio-Rad, Hercules, CA, USA). After the pulse-field gel electrophoresis (0.5× TBE, 14 °C, 200 V for 15 h with 60-s switching time, and for 8 h with a 90-s switching time), DNA was transferred to positively charged nitrocellulose membrane (GE Healthcare, Buckinghamshire, UK).

The number and chromosomal location of each *SUC* locus were determined by probing the membrane with DIG-labelled probes (Eurofins, Ebersberg, Germany). *S. paradoxus* and *S. cerevisiae* probes were designed according to the most conserved 5′ regions of *SUC2* gene.

Hybridization and detection reactions were carried out according to the Roche's DIG High Prime DNA Labeling and Detection Starter Kit 1 (Roche, MannHeim, Germany).

*S. paradoxus SUC2* probe sequence:

CGTCTGGGGTACGCCATTGTATTGGGGCCATGCTACTTCCGATGATTTGACCCACTGGCAAGACGAACCCATTGCTATTG

*S. cerevisiae SUC2* probe sequence:

ATGACAAACGAAACTAGCGATAGACCTTTGGTCCACTTCACACCCAACAAGGGCTGGATGAATGATCCAAATGG

### ddPCR for *SUC* copy number

The Southern blots of whole-chromosome pulsed-field gels could detect *SUC* loci in addition to *SUC2*. But because each chromosome has two telomeres, and because different chromosomal bands can colocalize on the gel, it cannot be used to precisely determine *SUC* copy number in strains that have subtelomeric copies of *SUC* in addition to *SUC2*. We therefore used droplet digital PCR (Bio-Rad QX100 system) to determine *SUC* copy number in the strains that had been determined by Southern blotting to contain multiple *SUC* loci, as well as in the 15 Chinese *S. cerevisiae* strains which we received most recently (as an alternative to Southern blotting). ddPCR uses simultaneous duplex reactions for target and reference genes within a single tube that contains about 20 000 reaction microdroplets, which are individually scored as positive or negative for the presence of amplicons by TaqMan fluorescence (see Huggett *et al.* 2013 for an introduction to the digital PCR technology). We used prevalidated TaqMan gene probes and primers designed by Life Technologies (CA, USA) for *SUC2* (VIC, Sc04134115_s1) and two reference genes *RPN5* (FAM, Sc04107686_s1) and *MNN1* (FAM, Sc04117288_s1).

We isolated genomic DNA (MasterPureTM Yeast DNA Purification Kit, Epicentre Biotechnologies) from C.Lab.1 as a single-copy control, C.Lab.2.*suc2::KANMX* as a zero copy control, the two strains identified by a previous study as having multiple *SUC* loci (C.Ginger.wine and C.Billi.wine; Naumov *et al.* 1996) as positive controls, as well as the wild strains to be tested (Please see Appendix S1, Supporting information). Genomic DNA was restricted with *Hin*dIII, as this enzyme has a conserved cut site within the *SUC2, RPN5* and *MNN1* open reading frames, but outside the binding regions of the TaqMan probes. In each case, 1 μg of genomic DNA, in 40-μL reactions, was digested using 5U of *Hin*dIII (BioLabs, New England) at 37 °C for 60 min, and terminated the reaction at 65 °C for 20 min. 2500 pg of restricted DNA, 10 μL of ddPCR SuperMix (Bio-Rad), 1 μL FAM reference probe/primer mixture (*RPN5* or *MNN1*), 1 μL VIC target probe/primer mixture (*SUC2*) were mixed and brought up to 20 μL final volume with molecular-grade water. Twenty microlitre reaction mixture and 70 μL droplet generation oil (Bio-Rad) were loaded into droplet generation cartridges, and ∼20 000 droplets were generated in separate wells. Droplet samples (∼40 μL) were transferred into the 96-well plates, and amplifications were carried out at 95 °C for 10 min, followed by 40 cycles of 94 °C for 30 s and 56 °C (optimized earlier by a thermal gradient PCR assay) for 1 min, and deactivated at 98 °C for 10 min. The plates were then loaded onto the QX100 droplet digital reader, and copy number was estimated using the quantasoft software (Bio-Rad). All ddPCR analyses were performed using two different 1-copy-reference-gene probes (*RPN5* and *MNN1*) on three independent DNA isolates (three biological replicates). Six data points were combined in the same graphic (see Fig. 2), as both probes gave a mixed copy number distribution, and the final results were given as mean copy number of all 6 data points.

### Variation in invertase production

Our screen of wild strains (see ‘Screening wild strains for invertase nonproducers’, above) was calibrated to detect the difference between producers and nonproducers of invertase. To precisely compare the invertase produced by ten producers with different copy numbers, we modified the assay to account for possible differences in cell density between the different strains. Strains were grown up as described before, but after resuspending each colony in 5 mL of sterile water, a 100-μL sample was taken and serially diluted to determine the cell density. We further optimized the dilutions of each strain to bring its OD_340_ measurement within the quantitative range. Thus, between 15 and 100 μL of the cell suspension from each strain was made up to the total test volume of 100 μL with a suspension of C.Lab.1.*suc2::KANMX* cells, prepared in the same way. This mixture was then assayed as previously, and the resulting signal was multiplied by this additional dilution factor. We converted absorbance to mg of invertase, using the standard curve in Appendix S4 (Supporting information), and we used the cell density to generate a per-cell measure of molecular invertase production for the standard laboratory producer strain (C.Lab.1), a selection of the wild *S. cerevisiae* strains from different sources (C.Oak.3, C.Soil.3, C.Cactus.1, C.Fruit.1, C.Cocoa.1), the three wild strains identified as having multiple *SUC* copies (C.Nectar.1, C.Nectar.2 and C.Nectar.3) and two domesticated control strains previously identified (Naumov *et al.* 1996) as having multiple *SUC* copies (C.Billi.wine and C.Ginger.wine). Every strain was tested three times to allow quantitative comparisons to be made between the strains (raw data are in Appendix S3, Supporting information).

### Determining the contribution of subtelomeric *SUC* loci to invertase production

We deleted the open reading frame of *SUC2* in the three wild strains identified as having multiple *SUC* copies (C.Nectar.1, C.Nectar.2, C.Nectar.3), and two domesticated control strains previously identified (Naumov *et al.* 1996) as having multiple *SUC* copies (C.Billi.wine, C.Ginger.wine). We used PCR-mediated gene replacement (Wach 1996) with the selectable drug resistance marker *NATMX* and the following PCR primers:

Forward primer for all strains:

CAAGCAAAACAAAAAGCTTTTCTTTTCACTAACGTATATGCGTACGCTGCAGGTCGAC

Reverse primer for C.Nectar.1, C.Nectar.2 and C.Nectar3:

CTTTTGAAAAAAATAAAAAAGACAATAAGTTTTATGACCTATCGATGAATTCGAGCTCG

Reverse primer for C.Ginger.wine and C.Billi.wine:

GCTTTTGAAAAAAATAAAAAGACAATAAGTTTTATAACCTATCGATGAATTCGAGCTCG

To test candidate transformants, we performed PCRs with two sets of diagnostic primers. Set 1 amplifies the region between upstream gene *YIL163C* and *SUC2* 5′ region. Set 2 amplifies the region between the 3′ end of *SUC2* and downstream gene *YIL161W*.

Primers used for diagnostic PCR are as follows:

Set 1 – upstream region:
  Suc1F CGATCCATTATGAGGGCTTC
  Suc1R GCCAAAAGGAAAAGGAAAGC
Set 2 – downstream region:
  Suc2F GAACATGACCACTGGTGTCG
  Suc2R GAGTTCCTTCGTTTCCCAAA

We also confirmed that *SUC2* was deleted from these five strains using the CHEF Southern blot (see Fig. S1, Supporting information). We then performed quantitative invertase production assays using the conditions described above (under ‘Variation in invertase production’) on the five wild-type strains (C.Nectar.1, C.Nectar.2 and C.Nectar.3; C.Ginger.wine and C.Billi.wine) and the five *SUC2* knockouts derived from them (C.Nectar.1.*suc2::NATMX*, C.Nectar.2.*suc2::NATMX*, C.Nectar.3.*suc2::NATMX,* C.Ginger.wine.*suc2::NATMX*, C.Billi.wine.*suc2::NATMX*, respectively). As mentioned before, three independent replicates were made of each assay, allowing quantitative comparisons to be made (raw data are on Appendix S3, Supporting information).

## Results

### No wild strains were invertase nonproducers

**Figure 1:**
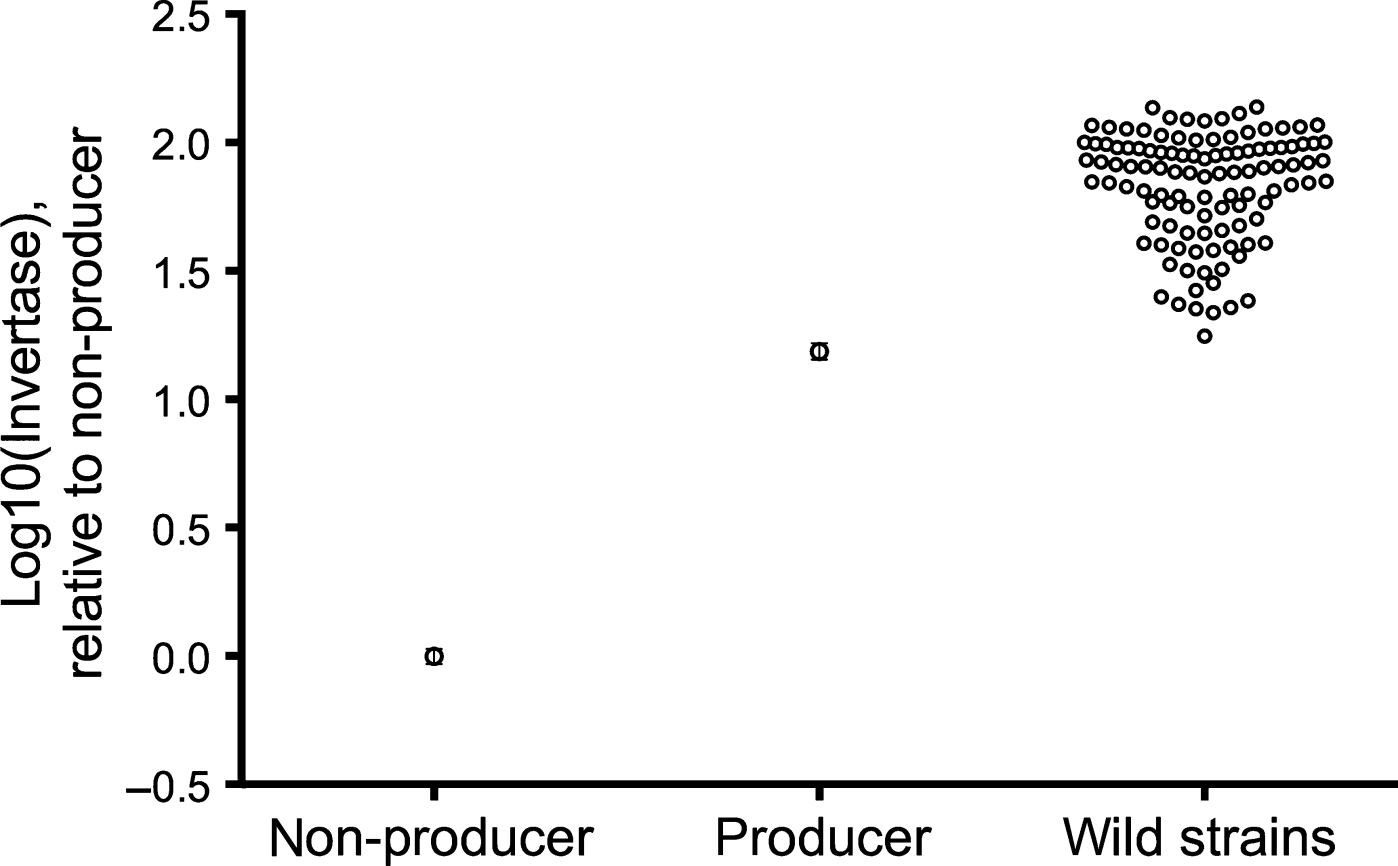
Screening of wild strains for invertase nonproducers. The invertase production of the 110 wild strains screened, as well as the standard laboratory invertase-producer strain C.Lab.1, is shown, relative to the production of the standard laboratory invertase nonproducer strain C.Lab.1.*suc2::KANMX*. All strains are described in Appendix S1 (Supporting information), and all data are listed in Appendix S2 (Supporting information).

Figure 1 shows that all the 110 wild strains that we tested produce more invertase than the standard invertase nonproducer or ‘cheater’ used in several previous experiments about cooperation (Greig & Travisano 2004; MacLean & Brandon 2008; Gore *et al.* 2009; MacLean *et al.* 2010). None of the 110 wild strains produced invertase at a level low enough to fall within the 95% confidence interval around the residual invertase activity of the standard nonproducer laboratory strain, C.Lab.1.*suc2::KANMX*. In fact, all the wild strains also had higher invertase activity than the 95% confidence interval around the invertase activity of the standard laboratory producer strain, C.Lab.1. We applied a Tukey post-hoc test to a one-way anova on these three groups and found that the 110 wild strains, as a group, produced significantly more invertase than both nonproducer and producer laboratory strains (*F*_2,113_ = 125.5, *P* < 0.0001).

### No *suc2* pseudogenes were detected in wild strains

Whole genome sequences existed for 29 *S. paradoxus* strains (Liti *et al.* 2009; Bergström *et al*. 2014). Consistent with their ability to produce invertase, we found intact open reading frames (ORFs) homologous to the reference *S. cerevisiae* strain (s288c/C.Lab.1) in all these strains. The length of the ORF was identical among all 29 *S. paradoxus* strains. Also for 8 *S. cerevisiae* strains, we found intact ORFs homologous to the reference strain (SGRP1: Liti *et al.* 2009; SGRP2: Bergström *et al*. 2014). There were no frameshift or nonsense mutations in any of the wild strains for which sequence was available (see Appendices S5 and S6, Supporting information for the *SUC2* nucleotide sequences identified in the wild strains used in this study).

### Three *S. cerevisiae* strains contained additional *SUC* genes

**Figure 2:**
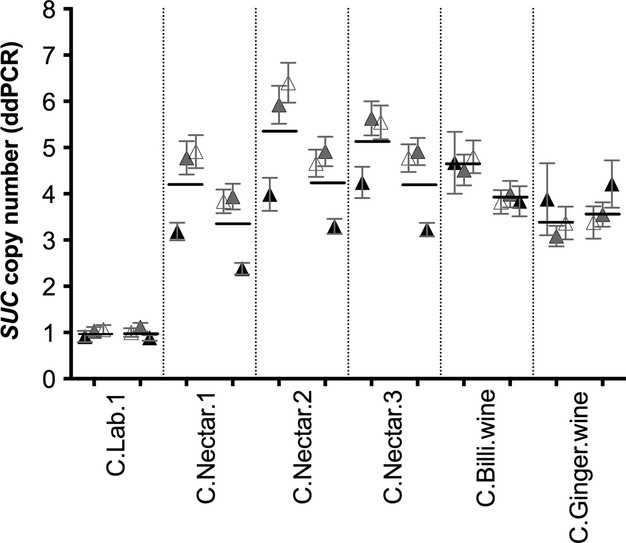
*SUC* gene copy number detection using droplet digital PCR (ddPCR) in five multilocus strains, normalized to a known single-copy control C. Lab.1 (first column). Three different symbol tones (dark, grey and empty) represent three different biological replicates. Copy number estimates calculated against *RPN5* reference probe are on the left-hand side of each column, and copy number estimates calculated against *MNN1* are on the right-hand side of each column. Black bars show the means of each set of three biological replicates.

Our Southern blots showed that all the wild *S. paradoxus* strains isolated from oak and maple trees contained just a single *SUC* locus, *SUC2*, located on chromosome IX. All 27 *S. cerevisiae* strains isolated from nature also contained *SUC2* on chromosome IX, but three *S. cerevisiae* strains (C.Nectar.1, C.Nectar.2 and C.Nectar.3) contained additional *SUC* loci on chromosome II (*SUC3*), on chromosome X (*SUC8*) and on chromosome XIV (*SUC9*) (Figs S1 and S2, Supporting information). ddPCR (Fig.2) shows that the *SUC* copy number of the three wild strains with multiple loci is closest to four, corresponding to one *SUC* open reading frame for each chromosome with a *SUC* locus (*SUC2,* plus the extra loci *SUC3, SUC8* and *SUC9*). All three of these wild strains were isolated from the same environment: Bertam palm (*Eugeissona tristis*) nectars in West Malaysia (Liti *et al.* 2009).

### Producers vary in their invertase production

**Figure 3:**
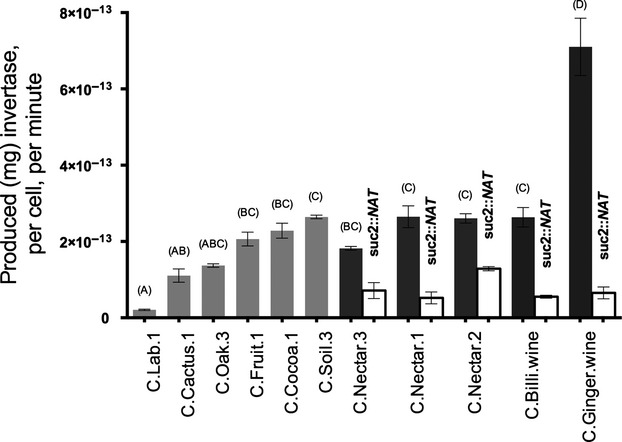
Light grey bars show the mean invertase production per cell for the single-copy standard producer and 5 other single-copy *S. cerevisiae* strains from different wild habitats. Dark grey bars show the production for the three strains with subtelomeric *SUC* loci isolated from Bertram palm nectar, and two strains from domesticated origins with subtelomeric loci as controls. Letters above the filled bars indicate which strains differ from each other with respect to their wild-type invertase production: strains with a letter in common are not significantly different. Open bars show the residual production of invertase from subtelomeric loci after *SUC2* was knocked out. Three replicate assays were made for each strain; error bars show the standard error of the mean.

We found that 11 different *S. cerevisiae* strains isolated from nine different domestic and wild environments varied significantly in their invertase production (Fig.3; *F*_10,22_ = 39.92, *P* < 0.0001). Post-hoc Tukey tests (letter above the bars in Fig.3) found that some, but not all, strains with four *SUC* copies produced significantly more invertase than strains with a single copy; some strains also produced significantly more invertase than other strains that had the same number of *SUC* copies. When grouped by number of *SUC* copies, the five strains with multiple *SUC* copies produced significantly more invertase than the six strains containing only *SUC2* (Student's *t*-test, *P* = 0.0023, *t* = 3.32, DF = 31), but this difference was driven by two strains (laboratory strain C.Lab.1 and domesticated strain C.Ginger.wine): when the analysis was repeated on the wild strains alone, no significant difference was detected between the strains with 1 *SUC* copy and the strains with four copies (Student's *t*-test, *P* = 0.0713, *t* = 1.8951, DF = 22). Thus, it was unclear whether or not additional subtelomeric copies of *SUC* contributed to the variation in invertase production, or whether it was caused simply by variation in *SUC2* expression. We therefore decided to test directly, by knocking out *SUC2*, whether the additional subtelomeric copies of *SUC* are expressed or whether they function only as silent backup copies for ‘cheats’.

### Subtelomeric *SUC* copies are not silent

Figure 3 shows that *SUC2* contributes much more to total invertase production than subtelomeric copies of *SUC*. Knocking out *SUC2* in the five strains with multiple copies reduces invertase production in every case, a statistically significant effect (*P* = 0.0312, paired sign test). The average reduction in invertase when *SUC2* was deleted was 64%, suggesting that each of three subtelomeric *SUC* genes contributes only about 12% to total invertase production. But subtelomeric copies are far from silent: the *SUC2* knockouts all produce more invertase than the standard laboratory producer strain C.Lab.1 (Fig.3).

## Discussion

Our results do not support the hypothesis that natural variation in *SUC* genes is caused by social conflict (Greig & Travisano 2004). It is more likely that different *SUC* genotypes are selected by habitats with different availabilities of sucrose (Naumov *et al.* 1996), but our survey does not contain enough variation for us to be certain.

### Invertase nonproducers

Our main aim was to determine whether invertase nonproducers existed in the same natural habitats as producers, which would be required in order for nonproducers to cheat. We found no nonproducers among the 65 oak-associated *S. paradoxus* strains that we tested nor among the 15 strains from maple trees. Unfortunately, the other habitats included in the survey had only a few strains available from each, so we might not find both producers and nonproducers cooccurring in the same type of habitat even if they were there. Nevertheless, we found no nonproducers at all among a total of 110 different wild strains (Fig.1). There is therefore no evidence to support the idea that nonproducing cheats occur among wild strains.

Our results stand in contrast to Naumov *et al*.'s (1996) finding of 11 nonproducers among a sample of 91 *S. cerevisiae* strains. One explanation is that Naumov *et al*. (1996) surveyed strains from a wider range of environments, which might select for or against the production of invertase according to sucrose adaptation hypothesis. Another is that most of Naumov's strains were associated with humans, whereas ours came only from natural sources. Artificial selection on domesticated species can increase diversity (Vila *et al.* 1999), as well as allowing loss of functions that would be important for survival in the wild (e.g. loss of pigmentation in domestic pigs and horses, Andersson & Georges 2004). It is therefore possible that invertase nonproducing mutants that would be eliminated by natural selection in the wild can persist by drift or even be selected in anthropogenic environments that are abundant in sugars other than sucrose or which lack producers as competitors. Thus, the variation observed in human-associated strains may be due to changes in environment, demography and population structure resulting from domestication. It is also possible that some domesticated environments produce conditions that allow cheating, for example by increasing population densities or environmental stability, compared to those conditions that would exist naturally, and thus, variation in domesticated strains could be due to the social conflict hypothesis. Because the evolutionary history of human-associated strains is obscure, it would be difficult to disentangle these explanations for the variation among domesticated yeast, but as there is no evidence for nonproducers and producers occupying the same habitat and abundant evidence for variability in sucrose availability, we, like Naumov *et al*. (1996), favour the sucrose adaptation hypothesis for domesticated strains as well as for the wild strains we describe here.

### Copy number variation

A secondary aim of the project was to determine whether variation in *SUC* copy number was consistent with social evolution.

According to the social conflict hypothesis as originally formulated (Greig & Travisano 2004), subtelomeric *SUC* loci could act as transcriptionally silenced backups which can be stochastically de-repressed (Gottschling *et al.* 1990; Louis 1995) or which could restore function to a *suc2* pseudogene by gene conversion (analogous to mating-type switching using silent telomeric copies of the hidden mating-type, HM, loci) (Naumov & Tolstorukov 1973; Hicks & Herskowitz 1977). Silent copies of *SUC* could allow cheats to switch back to invertase production when there are no cooperators to exploit, a form of ‘facultative cheating’ (Gore *et al.* 2009). This part of the social conflict hypothesis is now much less plausible because subsequent research has shown that subtelomeric silencing is predominantly a haploid phenomenon (Mercier *et al.* 2005). Indeed, the three wild strains we found with multiple *SUC* copies produced invertase at a high level, and they continued to do so even when *SUC2* was knocked out, showing that the remaining subtelomeric *SUC* loci are transcriptionally active and are not silenced backup copies (Fig.3). Further, all the strains with subtelomeric copies came from the same environment, Bertram palm nectar, and all strains from this environment contained three subtelomeric *SUC* alleles in addition to *SUC2*: there was no genotypic variation within the environment as predicted by the social conflict hypothesis. This could simply be because our tiny sample contained only three strains, but it is also most consistent with the sucrose adaptation hypothesis. Sucrose is the major carbon source in most plant nectars (Corbet 2003; Pacini *et al.* 2003; Dupont *et al.* 2004; Wiens *et al.* 2006; Peay *et al.* 2012), and Bertam palm nectars contain high and stable concentrations of sucrose (∼10%; Wiens *et al.* 2006). However, these strains are very closely related: C. Nectar.1 differs from C.Nectar.2 by just 0.0059% of nucleotides across the whole genome, and from C. Nectar.3 by 0.019%; C.Nectar.2 and C.Nectar.3 differ by 0.012% (Liti *et al.* 2009). Given the small sample size, the high genetic relatedness and the likelihood that all three strains inherited their subtelomeric *SUC* genes by common descent, we cannot exclude the possibility that the expansion of the *SUC* gene family in this environment is due to neither social evolution nor environmental selection, but simply genetic drift.

The expression of invertase from strains with subtelomeric *SUC* loci shows that they are not ‘cheats’. However, a social model could still be used to explain their evolution if the originally proposed roles of cooperator and cheat were reversed. If strains with more *SUC* copies produce more invertase, they could be considered cooperators instead of cheats, and they could feed other, cheating, strains that have only *SUC2* and produce less. As under the original social explanation for *SUC* genetic variation, we would predict that cheats and cooperators should occur in the same environment. Whilst we might not expect to detect such copy number variation among only three strains from Bertram palm nectar, we would expect to find variation within the well-sampled oak-tree and maple-tree habitats, but we did not. Instead, we find copy number variation between (but not within) environments that differ in sucrose availability. Whilst we must be cautious not to overgeneralize from just three closely related strains, the little copy number variation we do find in our survey is clearly better explained by the sucrose adaptation hypothesis than by the social conflict hypothesis.

### Is invertase production a cooperative trait?

We previously proposed the social conflict hypothesis to explain variation in *SUC* genotypes among *S. cerevisiae* strains (Greig & Travisano 2004). But because *S. cerevisiae* is domesticated, and isolates came from many different sources, it was difficult to know whether different genotypes evolved in a common environment that would permit social cheating. In this survey of wild strains, we find very little variation of *SUC* genotypes, and the limited variation we do find occurs between, and not within, environments. The genetic variation is therefore better explained by adaptation to different environmental levels of sucrose than by social conflict. However, given the lack of variation in our samples, we have very limited power to differentiate between the two hypotheses. The ideal survey would test the invertase production and the *SUC* genotype of multiple strains isolated from at last two different natural habitats that differed in their sucrose availability. Such a design would have the best chance of being able to definitively distinguish the difference between the two hypotheses explaining variation for *SUC*. If different *SUC* genotypes are selected by the local availability of sucrose, then the two environments will be fixed for different genotypes. If social conflict produces variation, then we would expect more variation within the high-sucrose environment than within the low-sucrose environment. Unfortunately, such well-sampled natural habitats differing in sucrose availability do not exist, but we hope that as research in yeast natural history progresses, such a survey may be possible in the future.

Authors have previously cited the variation in *SUC* genotypes as evidence that cheating occurs in nature (Greig & Travisano 2004; MacLean & Brandon 2008; Gore *et al.* 2009), but here we show that the evidence has been misinterpreted. This has significant consequences for the use of invertase production as an experimental model of cooperation. Cooperative traits are properly defined not merely as those traits that benefit others, which would be nonsensically overinclusive, but those traits that *evolved because* of the benefits they convey to others (West *et al.* 2007). Thus, it is important to show that cooperation occurs in the environment in which a putative cooperative trait evolved, and the existence of natural genetic variation was presented as evidence that invertase production evolved in nature as a cooperative trait. It is worth noting as an aside, though, that the existence of natural cheats is not sufficient to prove a trait as cooperative: we would not consider scatter-hoarding of nuts by squirrels to be a cooperative trait, even though hoarded nuts are often eaten by scroungers and not by the squirrel that buried them (Stapanian & Smith 1978). To prove that invertase production evolved as a cooperative trait, one would need to show that not only that social conflict over invertase sharing occurs in nature, but also that invertase sharing was actually selected. Surveys like ours cannot therefore determine whether or not invertase production is a cooperative trait. Even if the natural variation for *SUC* copy number is not caused by social conflict, social conflict may nonetheless underlie other forms of genetic variation for invertase production (for example, Fig.3 shows there is considerable and significant variation in invertase production even among strains containing only *SUC2*). And even if social conflict does not cause any natural genetic variation in invertase production, it is still possible that invertase production evolved as a cooperative trait in nature. And even if it did not evolve as cooperative trait in nature, invertase sharing in an experimental setting could still be a useful model for cooperation. We are mindful, though, of the words of G.C. Williams: ‘Adaptation should be attributed to no higher a level of organization than is demanded by the evidence’ (Williams 1996). In our opinion, a trait should not be called cooperative until more parsimonious explanations for its evolution have been rejected.

## Data accessibility

The available *SUC2* nucleotide sequences, in FASTA format, for 29 *S. paradoxus* and 8 *S. cerevisiae* strains are accessible on Appendices S5 and S6 (Supporting information), respectively. Raw data for all invertase assay experiments, including absorbance reads and calculations, are accessible on Appendices S2, S3 and S4 (Supporting information).

## Supporting Information

Appendix S1 List of all strains used in this study with strain names and details.

Appendix S2 Invertase producer screening results for each wild strain, producer strain, and nonproducer strain. Wild strains were tested once; control strains were tested as 3 replicates.

Appendix S3 Invertase assay result for 11 strains and 5 *suc2* knockout strains. Three measurements were performed for each strain.

Appendix S4 Invertase assay results performed using commercial invertase enzyme to determine the linear response range and to calculate the invertase production using the equation of the linear regression (*R*^2^ = 0.948, six data points, three replicates each). None of our measurements gave more glucose reading over the top point of this linear regression line.

Appendix S5 *S. paradoxus SUC2* nucleotide sequences available for 29 strains.

Appendix S6 *S. cerevisiae SUC2* nucleotide sequences available for eight strains. Sequence files include ∼130 base pairs upstream and 200 base pairs downstream of the start and the stop codons, respectively.

Fig S1 Southern blot confirmation of *SUC2* knockouts in the multiple-copied strains, and common laboratory strain C.Lab.1 (S288c).

Fig S2 CHEF gel and corresponding Southern blot assay showing four different chromosomal locations of *SUC* genes in C.Nectar.1, C.Nectar.2. and C.Nectar.3 strains. This CHEF gel was ran longer (0.5× TBE, 14 °C, 200 V for 30 h with 60-s switching time, and for 12.5 h with a 90-s switching time) to further separate chromosome II, XIV, X bands.
