# Supplementary material for: The genetics of a putative social trait in natural populations of yeast
Source: Mol Ecol. 2014 Oct 4;23(20):5061–71. doi: 10.1111/mec.12904 (PMC4285311; doi:10.1111/mec.12904)
Supplement: Fig S1 — Southern blot confirmation of SUC2 knockouts in the multiple-copied strains, and common laboratory strain C.Lab.1 (S288c). [file mec0023-5061-SD7.pdf]

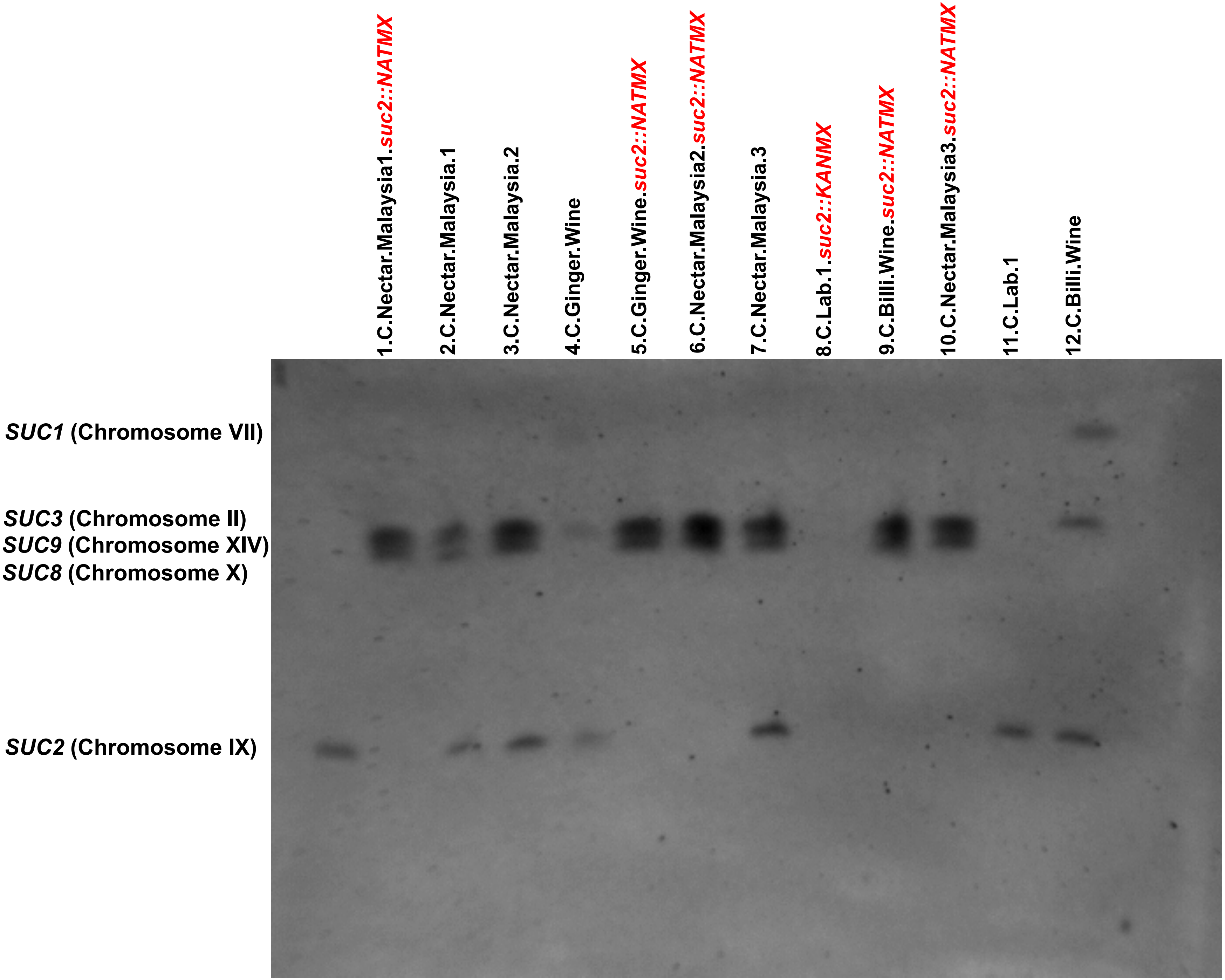

1.C.Nectar.Malaysia1.suc2::NATMX
2.C.Nectar.Malaysia.1
3.C.Nectar.Malaysia.2
4.C.Ginger.Wine
5.C.Ginger.Wine.suc2::NATMX
6.C.Nectar.Malaysia2.suc2::NATMX
7.C.Nectar.Malaysia.3
8.C.Lab.1.suc2::KANMX
9.C.Billi.Wine.suc2::NATMX
10.C.Nectar.Malaysia3.suc2::NATMX
11.C.Lab.1
12.C.Billi.Wine
SUC1 (Chromosome VII)
SUC3 (Chromosome II)
SUC9 (Chromosome XIV)
SUC8 (Chromosome X)
SUC2 (Chromosome IX)
