# Supplementary figures and images for: The genetics of a putative social trait in natural populations of yeast
Source: Mol Ecol. 2014 Oct 4;23(20):5061–71. doi: 10.1111/mec.12904 (PMC4285311; doi:10.1111/mec.12904)

*SUC3* (Chromosome II)  
*SUC9* (Chromosome XIV)  
*SUC8* (Chromosome X)

*SUC2* (Chromosome IX)

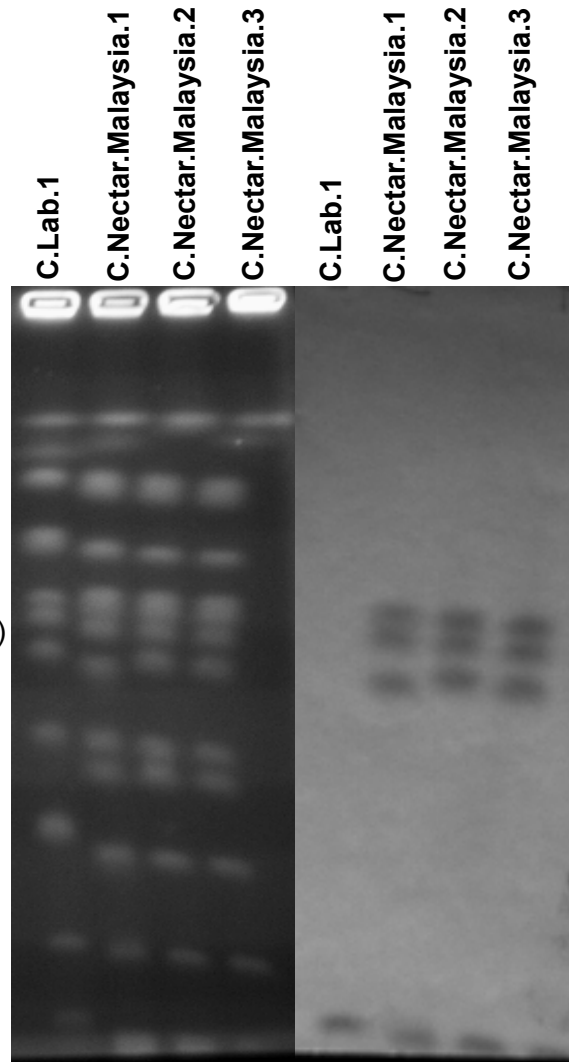

Supplement: Fig S2 — CHEF gel and corresponding Southern blot assay showing four different chromosomal locations of SUC genes in C.Nectar.1, C.Nectar.2. and C.Nectar.3 strains. This CHEF gel was ran longer (0.5× TBE, 14 °C, 200 V for 30 h with 60-s switching time, and for 12.5 h with a 90-s switching time) to further separate chromosome II, XIV, X bands. [file mec0023-5061-SD9.pdf]
